# SPINNAKER: an R-based tool to highlight key RNA interactions in complex biological networks

**DOI:** 10.1186/s12859-022-04695-x

**Published:** 2022-05-06

**Authors:** Paola Paci, Giulia Fiscon

**Affiliations:** 1 Department of Computer, Control and Management Engineering “Antonio Ruberti” (DIAG), Sapienza University of Rome, Rome, Italy; 2 Institute for Systems Analysis and Computer Science “Antonio Ruberti”, National Research Council, Rome, Italy

**Keywords:** Algorithms, ceRNA network, Network theory, miRNA sponge

## Abstract

**Background:**

Recently, we developed a mathematical model for identifying putative competing endogenous RNA (ceRNA) interactions. This methodology has aroused a broad acknowledgment within the scientific community thanks to the encouraging results achieved when applied to breast invasive carcinoma, leading to the identification of PVT1, a long non-coding RNA functioning as ceRNA for the miR-200 family. The main shortcoming of the model is that it is no freely available and implemented in MATLAB®, a proprietary programming platform requiring a paid license for installing, operating, manipulating, and running the software.

**Results:**

Breaking through these model limitations demands to distribute it in an open-source, freely accessible environment, such as R, designed for an ordinary audience of users that are not able to afford a proprietary solution. Here, we present SPINNAKER (SPongeINteractionNetworkmAKER), the open-source version of our widely established mathematical model for predicting ceRNAs crosstalk, that is released as an exhaustive collection of R functions. SPINNAKER has been even designed for providing many additional features that facilitate its usability, make it more efficient in terms of further implementation and extension, and less intense in terms of computational execution time.

**Conclusions:**

SPINNAKER source code is freely available at https://github.com/sportingCode/SPINNAKER.git together with a thoroughgoing PPT-based guideline. In order to help users get the key points more conveniently, also a practical R-styled plain-text guideline is provided. Finally, a short movie is available to help the user to set the own directory, properly.

**Supplementary Information:**

The online version contains supplementary material available at 10.1186/s12859-022-04695-x.

## Background

microRNAs (miRNAs) are single-stranded short RNAs (about 20–22 nucleotide) that post-transcriptionally regulate gene expression by degradation or translation inhibition of their target messenger RNAs (mRNAs). Most of the biological processes have been shown to entail the regulation orchestared by miRNAs, such as cell proliferation, differentiation, metabolism, development, and apoptosis [1]. A novel mechanism of miRNA regulation regarding the ability of RNAs to compete for the miRNA binding has recently been discovered [2, 3]. Key triggers of this new layer of post-transcriptional regulation are the so-called competing endogenous RNAs (ceRNAs)—or miRNA ‘sponges’, involving both coding and non-coding RNAs, such as pseudogenes [2], circular RNAs [4, 5], and long non coding RNAs (lncRNAs) [6, 7]. ceRNAs exert their decoy activity by recruiting miRNA molecules through base-pairing with miRNA-recognition elements (MREs) that they share with a target, consequently determining the target release from the miRNA control. The ceRNA cross-talks (i.e., their interactions mediated by miRNAs) have been identified as drivers of most of the pathological conditions, including human cancers [2, 8–10].

The research field related to ceRNA mechanism has rapidly grown during the last decade, as observed by looking at the increasing number of published studies over the last years (Additional file 1: Figure S1). Meanwhile, the recent years have seen the development of several computational methodology to build ceRNA networks. Yet, there remain great opportunities as well as challenges to propose computational models helping the generation of hypotheses able to drive wet-lab experiments towards the elucidation of the roles of ceRNAs in a particular disease [11].

Since considering all types of miRNA sponges can lead to a high computation complexity, in our recent study we focused on lncRNAs given their acknowledged importance in diverse biological and physiopathological contexts [12] and we developed a new methodology suitable to exploring their potential role as ceRNA regulators [13]. According to a recent review, comparing the most widespread computational models for ceRNA-ceRNA interactions’ identification [11], our method proved to be the best in terms of the percentage of predicted RNAs acting as ceRNAs related to breast invasive carcinoma. However, the main limitation towards its broader usability is that it was no freely distributed so far, and originally written in MATLAB, a proprietary programming language requiring a paid license to install, exploit, operate, and run the software. The interesting and acknowledged results obtained by applying our model to breast cancer and the necessity to spread it to a broader scientific audience created the call to handle this issue and to design an open-source version of the model for a universal community of non-expert users.

Here, we present SPINNAKER, the implementation of the originally developed ceRNA model [13] in a exhaustive collection of R functions. Yet, SPINNAKER comes as a simplified and an improved version with respect to the original MATLAB-based implementation (Additional file 2), ensuring more efficiency by speeding up the entire pipeline of many orders of magnitude (i.e., from many hours to minutes). In particular, an additional feature of SPINNAKER is the possibility of choosing among different pools of RNAs acting as ceRNAs, as long as the total number of triplets to be tested is within the order of magnitude of ${10}^{6}$, otherwise it collides with a huge computation complexity.

### Implementation

**Fig. 1:**
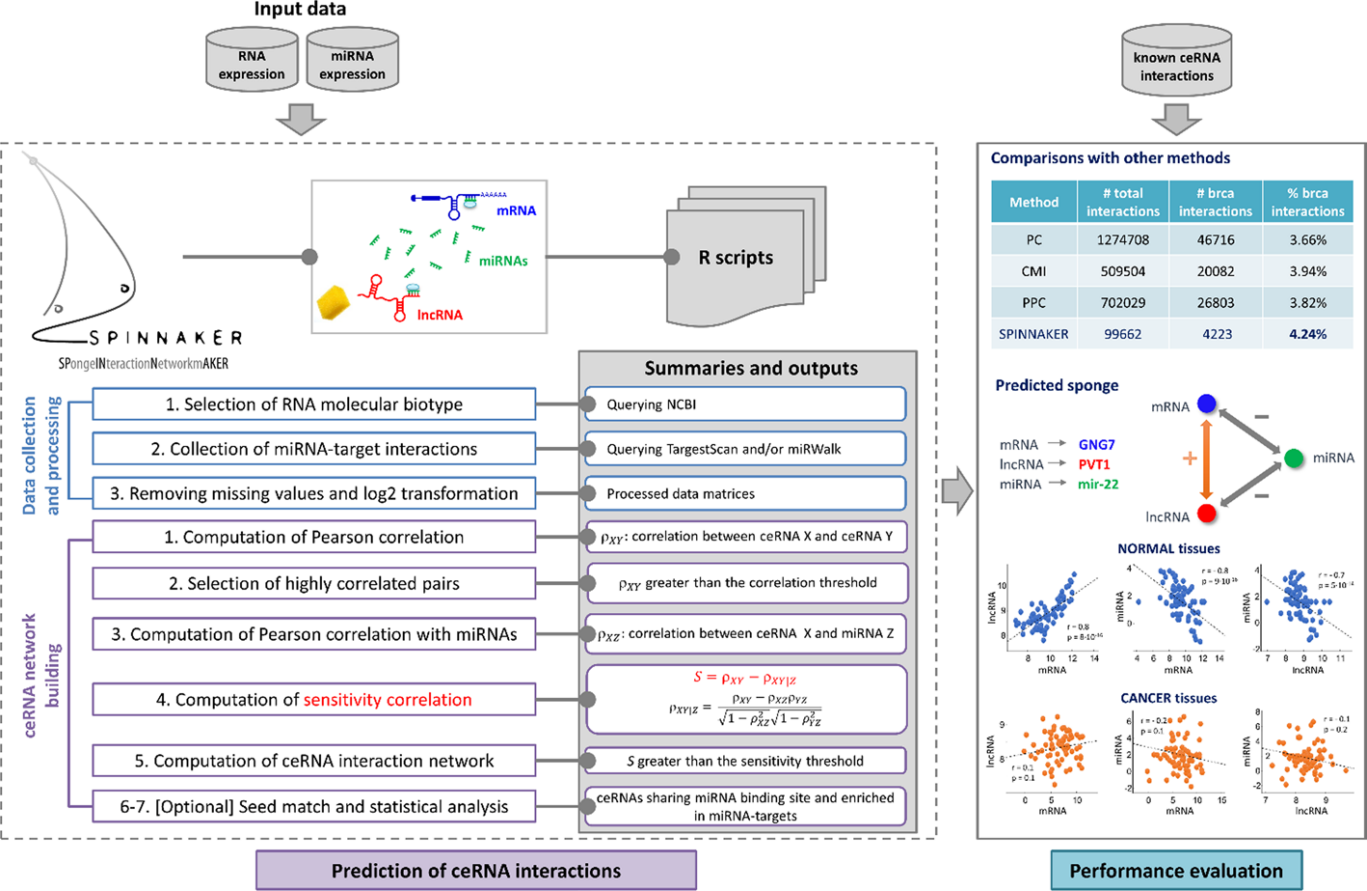
SPINNAKER conceptual organization. [Left] Algorithm steps. [Top right] Comparison of ceRNA predictions obtained by SPINNAKER, when applied to breast cancer dataset [13], with respect to other statistics-based methods in identifying experimentally confirmed and breast cancer related ceRNA interactions, defined as those in which the two interacting ceRNAs are breast cancer related genes [11]. [Bottom right] Example of ceRNA interactions predicted by SPINNAKER in breast normal tissues from breast cancer dataset [13]. Scatter plots of the ceRNA expression levels (log2-scale) in normal (blue dots) and cancer (orange dots) tissues; r = Pearson correlation coefficient, p = p-values

SPINNAKER (SPongeINteractionNetworkmAKER) is an R-based implementation of a methodology for identifying putative ceRNA interactions that we recently published along with its application in breast invasive carcinoma [13]. SPINNAKER takes as input normalized expression levels of RNAs and miRNAs (e.g., FPKM) and predicts the ceRNA interaction network by implementing two modules: 1) data collection and processing, 2) ceRNA network building (Fig. 1). Each module consists of several steps detailed in the following.

#### Module 1: data collection and processing

The goal of this module is to collect and process data for running SPINNAKER.

(i) Selection of RNA molecular biotype
  To define the molecular entities competing for the miRNA binding, SPINNAKER automatically queries NCBI's Gene database, including information about chromosomal localization, nomenclature, gene products, and their attributes (e.g., molecular biotype). Then, SPINNAKER separates the two selected classes of candidate ceRNAs to be tested (e.g., protein coding versus long non-coding RNAs).
(ii) Collection of miRNA-target interactions
  SPINNAKER collects miRNA sequences from miRBase (currently release 22.1, October 2018, http://www.miRBase.org). Then, it retrieves the predictions of miRNA-mRNA target interactions from TargetScan [14] and the predictions of miRNA-lncRNA target interactions from miRWalk [15]. TargetScan appears as the most up-to-date database for sequence-based predictions of miRNA-target interactions, it predicts miRNAs targets by considering the exact matching between the seed region of a miRNA (i.e., positions 2–7 from the miRNA 5′-end) and the 3′ UTR of its targets [16]. miRWalk provides the predicted and validated miRNA-binding sites of known genes of human and other species, most importantly including lncRNAs-miRNA interactions, and it is entirely updated more than once a year. miRWalk core is based on TarPmiR [17], a prediction tool that exploits an approach based on random-forest algorithm to look for putative miRNA binding sites within the whole transcript sequence including the 3′-UTR, 5′-UTR, and CDS [15].
(iii) Removing missing values and log2 transformation
  SPINNAKER applies a logarithmic (log2) transformation to the RNAs and miRNAs expression levels and conducts a processing analysis to remove those genes having too many missing values among the samples (i.e., by default, SPINNAKER filters out entries showing missing values for more than ten percentage of samples).

#### Module 2: ceRNA network building

The goal of this module is to build the ceRNA interaction network.

**Fig. 2:**
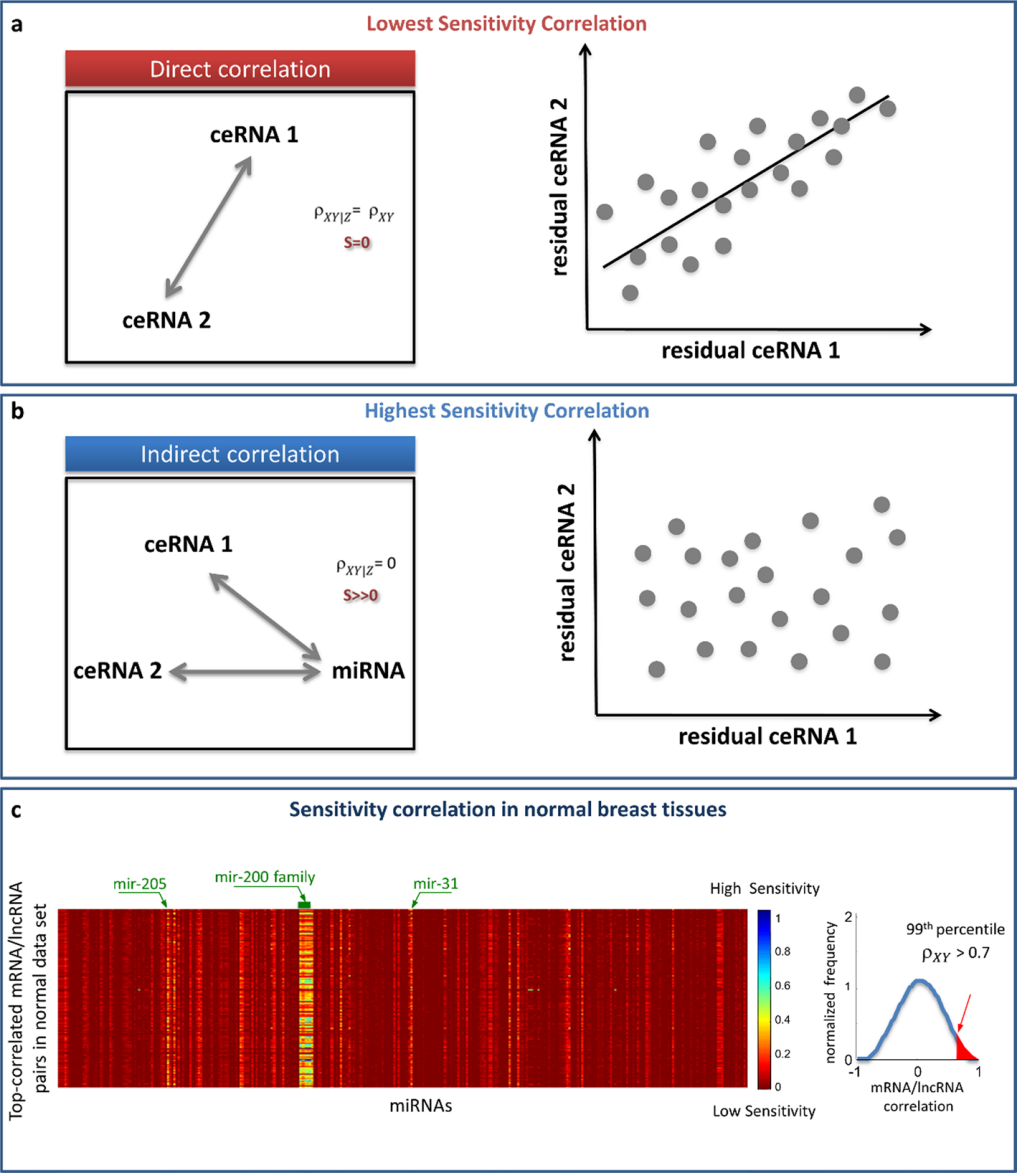
Sketch of sensitivity correlation measure. **a**, **b** The *sensitivity correlation* (S) is the difference between the Pearson correlation (${\rho }_{XY}$) and the partial correlation (${\rho }_{XY|Z}$). Two extreme situations are reported: **a** the correlation is direct and the miRNA is not mediating the interaction; **b** the correlation is undirected and the miRNA is mediating the interaction. In the first case, ${\rho }_{XY|Z}= {\rho }_{XY}$, S = 0, and, since Z is not explaining anything, the residuals are highly correlated. In the second case ${\rho }_{XY|Z}=0$, S is maximum, and, since Z is explaining all the variability, no correlation is found between residuals. (c) Heatmap of S, calculated for the top-correlated RNA pairs (i.e., showing ${\rho }_{XY}$ > 0.7) in the normal breast dataset [13]. Bright vertical stripes refer to a small set of miRNAs mediating the interactions between the top-correlated RNA pairs; S values increases from red (S = 0) to blue (S = 1)
  An example of output of this step, which describes the sensitivity correlation obtained for normal breast tissues [13], is presented in Fig. 2c. The computed sensitivity matrix is rendered as an heatmap, where rows represent the highly correlated RNA pairs, columns refer to all the analysed miRNA, and sensitivity values are color-coded increasing from red to blue.
  The sensitivity correlation calculated in normal breast samples [13] unveiled a general trend of the interactions between RNA pairs marked by highly positive correlations that appear miRNA-independent (S∼0, red background in Fig. 2c), except for a small pool of miRNAs for which the sensitivity appears to be pretty different from zero (bright vertical stripes in Fig. 2c). This observation highlights the presence of particular miRNAs (such as miR-200 family) able of generating a crosstalk throughout the whole transcriptome. These miRNAs, corresponding to the bright vertical stripes in the sensitivity heatmap, represent the links of ceRNA network generated by SPINNAKER, whereas the highly correlated RNA pairs with high sensitivity correlation mediated by these miRNAs represent the nodes of the ceRNA network.
  It is worth noting that the outcome of SPINNAKER might not be rendered as a single heatmap, since the size of the picture could exceed the memory limits of computer. To overcome this limitation, SPINNAKER segregates the sensitivity matrix into a variable number of heatmaps (depending on the total number of RNA pairs), each one composed of a maximum number of 5000 rows (RNA pairs).
(v) Computation of ceRNA interaction network.
  SPINNAKER selects the XYZ triplets with S greater than a defined threshold (by default equal to the 99^th^ percentile) on the overall distribution of the S-values. This threshold allows to reduce both the computational effort in evaluating the ceRNA interactions and the number of false positives. The X and Y variables correspond to the top-correlated RNA pairs. Then, SPINNAKER builds the ceRNA interaction network.
  Nodes in the ceRNA network represent ceRNAs marked by a high correlation between their expression profiles; whereas links represent miRNAs that are mediating their interactions. A link between two nodes (ceRNAs) occurs if they fulfilled the following conditions (Fig. 3): (1) showing a high Pearson correlation value; (2) showing a high sensitivity correlation value.

(i) Computation of Pearson correlation
  SPINNAKER computes the Pearson correlation coefficients between the expression profiles of the RNA pairs $({\rho }_{XY})$.
(ii) Selection of highly correlated pairs
  SPINNAKER selects the RNA pairs with ${\rho }_{XY}$ greater than a defined threshold (by default equal to 99th percentile) on the overall correlation distribution. This threshold allows to reduce both the computational effort in evaluating the RNA interactions and the number of false positives.
(iii) Computation of Pearson correlation with miRNAs
  SPINNAKER computes the Pearson correlation coefficient between the expression profiles of RNA X and miRNA Z (${\rho }_{XZ}$) and the expression profiles of RNA Y and miRNA Z (${\rho }_{YZ}$).
(iv) Computation of sensitivity correlation
  To determine if the Pearson correlation between the RNA pairs is direct or mediated by the miRNA, SPINNAKER implements the following metric, called *sensitivity correlation* S (Fig. 2):
  $$S={\rho }_{XY}-{\rho }_{XY|Z}$$
  with ${\uprho }_{XY}$ referring to the Pearson correlation coefficient between RNA X and RNA Y, and ${\uprho }_{XY|Z}$ referring to the partial correlation between RNA X and RNA Y controlling for the miRNA Z defined as:
  $${\rho }_{XY|Z}= \frac{{\rho }_{XY}-{\rho }_{XZ}{\rho }_{YZ}}{\sqrt{1-{\rho }_{XZ}^{2}}\sqrt{1-{\rho }_{YZ}^{2}}}$$
  with ${\rho }_{XZ}$ (${\rho }_{YZ}$) referring to the Pearson correlation between RNA X (RNA Y) and miRNA Z [18]. The partial correlation ${\rho }_{XY|Z}$ measures how much the correlation between two variables X (RNA 1 expression profile) and Y (RNA 2 expression profile) remains after removing a third variable Z (miRNA expression profile). Thus, a low value of sensitivity correlation (i.e., partial correlation approaching the Pearson correlation) refers to a direct interaction between the two RNAs competing for the same miRNA, i.e., whose interaction is not mediated by the miRNA (Fig. 2a); while a high value of sensitivity correlation (i.e., partial correlation approaching to zero) refers to an indirect interaction between the two RNAs competing for the same miRNA, i.e., whose interaction is mediated by the miRNA (Fig. 2b).

**Fig. 3:**
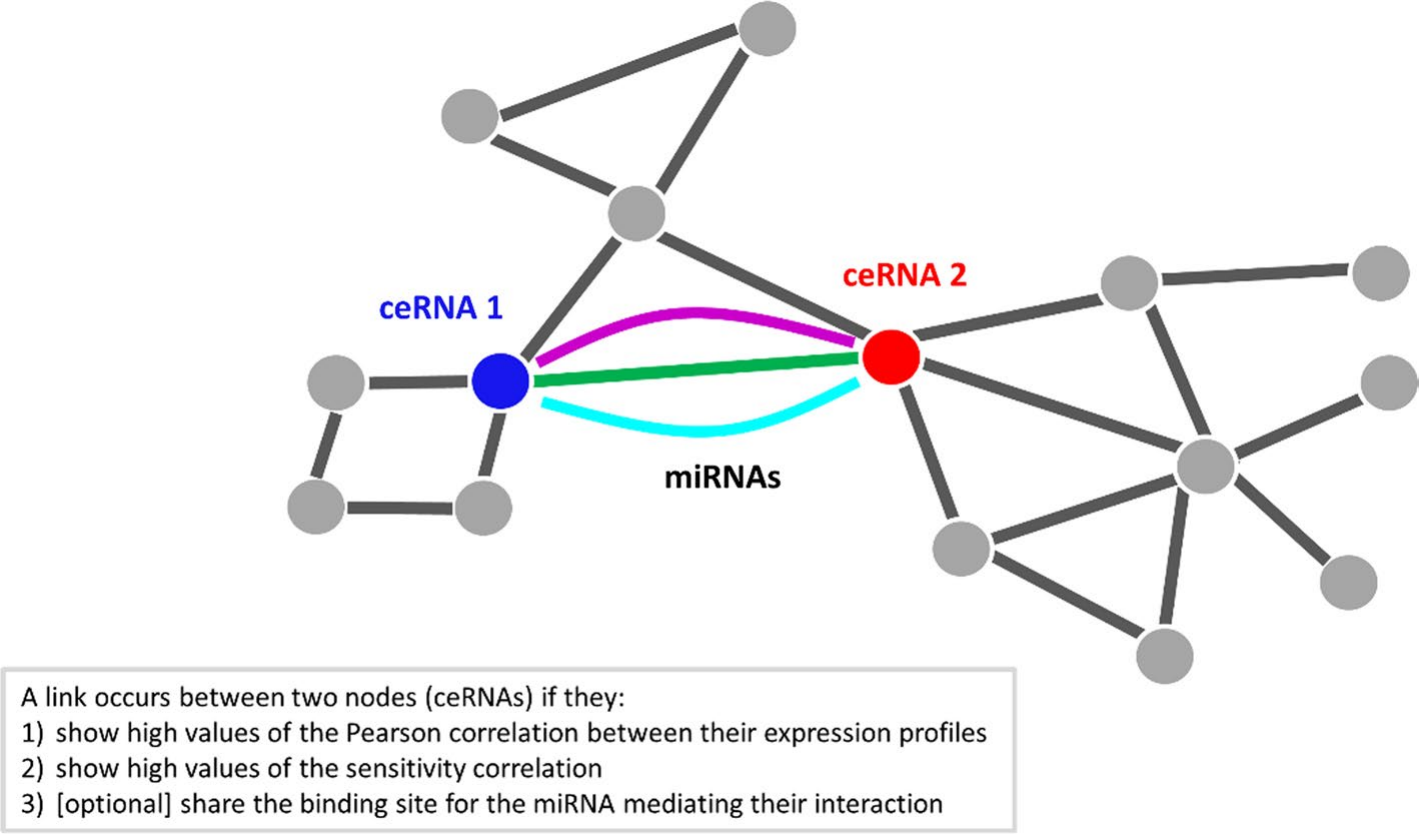
Sketch of ceRNA interaction network. Nodes in this network represent ceRNAs competing for the miRNA binding (e.g., mRNAs and lncRNAs); whereas links represent miRNAs mediating their interaction
(vi) [Optional] Search seed-match for all triplets
  SPINNAKER searches for the seed-match of all the highly correlated pairs with the miRNA mediating their interactions, in order to narrow the above selected triplets (step v.) to those including only ceRNAs that are targets of the shared miRNA.
(vii) [Optional] Computation of statistical analysis
  For each miRNA, SPINNAKER performs a seed-match enrichment analysis by computing the following statistics (i.e., p-value resulting from the hypergeometric test) (Fig. 4):
  $$p=1-\sum_{i=0}^{X-1}\frac{\left(\genfrac{}{}{0pt}{}{K}{i}\right)\left(\genfrac{}{}{0pt}{}{U-K}{S-i}\right)}{\left(\genfrac{}{}{0pt}{}{U}{S}\right)}=\sum_{i=X}^{S}\frac{\left(\genfrac{}{}{0pt}{}{K}{i}\right)\left(\genfrac{}{}{0pt}{}{U-K}{S-i}\right)}{\left(\genfrac{}{}{0pt}{}{U}{S}\right)}$$
  where U is the universe dimension, that is the number of the top-correlated RNA pairs; K is the property, that is the number of RNA pairs sharing the binding site for the miRNA under test; S is the selection, that is the number of RNA pairs with high sensitivity for the miRNA under test; X is the number of RNA pairs with sensitivity correlation exceeding the defined threshold on the S-values distribution and sharing the binding site for the miRNA under test.

**Fig. 4:**
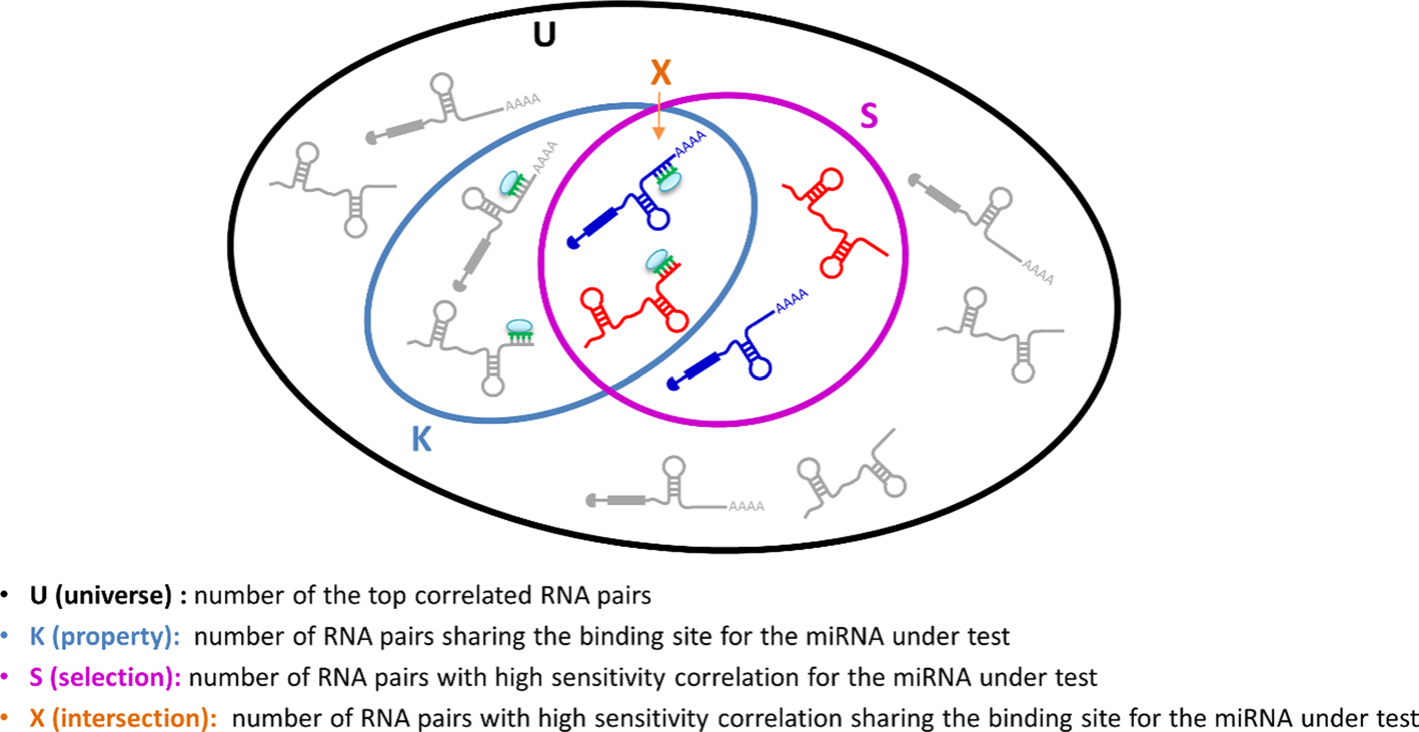
Sketch of hypergeometric test conducted for the seed-match analysis. For a given miRNA, the figure shows how the ensembles are chosen to compute the hypergeometric test
  The output of this step is the ceRNA interaction network with the additional information of p-values, and where a link between two nodes (ceRNAs) occurs if they fulfilled the following conditions (Fig. 3): (1) showing a high Pearson correlation value; (2) showing a high sensitivity correlation value; and (3) sharing the binding sites for the miRNAs mediating their interaction.
  An example of output, which describes the ceRNA network built for normal breast tissues [13], is presented in Fig. 5. The ceRNA network released by SPINNAKER can be easily uploaded on Cytoscape software [19], an open source tool for complex networks visualization and integration with other types of attribute data. By using Cytoscape, the ceRNA network can be visualized by adding a different color to each miRNA and analyzed by using several built-in apps, like the “network analyzer” tool. In this way, the user can obtain the total number of connected components of the ceRNA network and other network properties associated to each node, such as betweenness, closeness, clustering coefficient, degree, and thus consequently identified the network hubs (i.e., nodes with more than 5 links [20]).

**Fig. 5:**
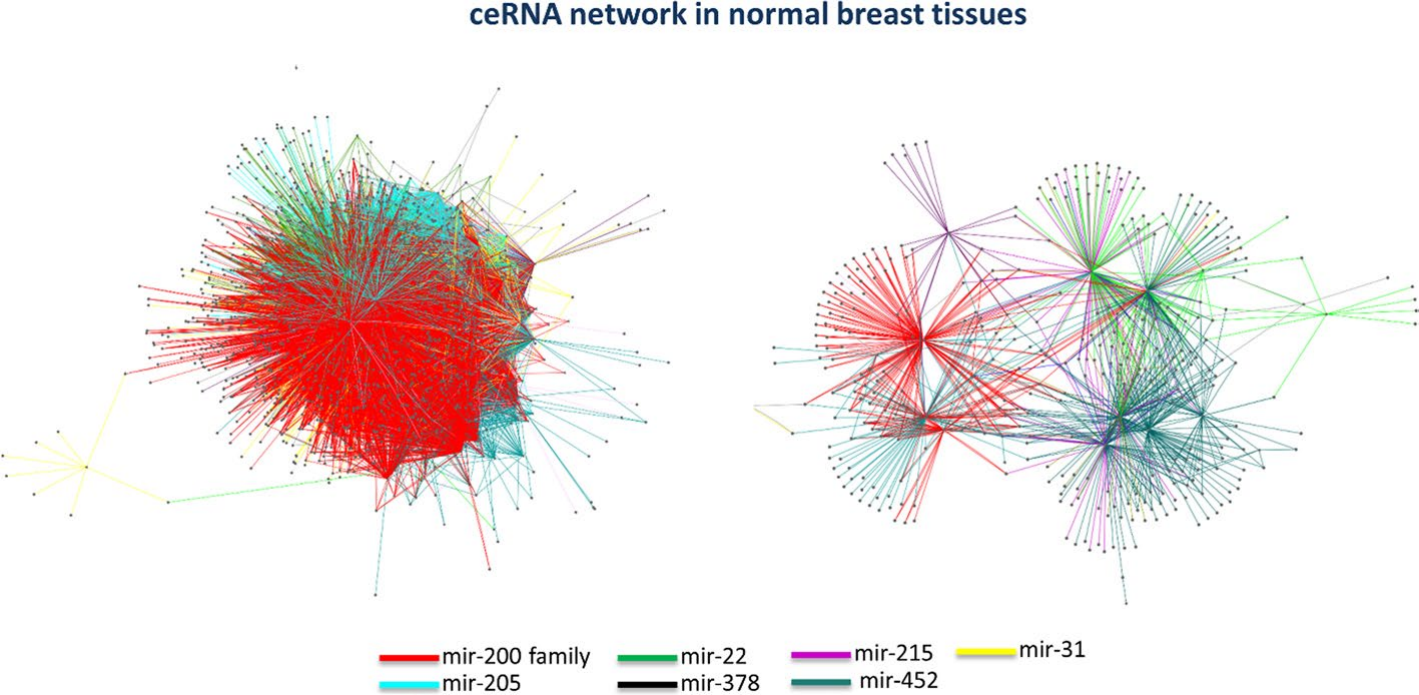
Example of ceRNA interaction network. ceRNA network constructed from the gene expression data of normal breast tissues in [13] and visualized by using Cytoscape [19]
  In particular, the ceRNA network of normal breast (32,375 links and 1738 nodes) is markedly distinguished into two well-connected components: a smaller one (954 links and 378 nodes) mainly dominated by miR-452 whose mRNAs functioning as ceRNAs were functionally enriched in cellular metabolic processes, and a larger one (31,417 links and 1354 nodes) mainly controlled by the miR-200 family and functionally enriched in cell–cell adhesion functions [13]. Of note, the first hub of ceRNA normal breast network was the lncRNA PVT1, revealing a net binding preference with the miR-200 family and competing with mRNAs mostly associated to cancer development and progression (e.g., CDH1, GATA3, RUNX1, RUNX3, TP53, TP63, TP73).

## Results

### Comparisons with other methods

The widespread computational approaches developed for modelling ceRNA regulatory mechanism in human cancers can be grouped into two main classes: (i) statistics-based methods, which exploited statistical analyses (e.g., multivariate analysis) to infer putative evidences of ceRNA crosstalk and construct ceRNA interaction networks by considering miRNA expression levels (Additional file 1: Table S1); (ii) mathematical methods, which make use of stochastic or deterministic models to predict and analyze the behavior of ceRNA crosstalk (Additional file 1: Table S2). The model implemented by SPINNAKER, based on sensitivity metric, is included in the statistics-based methods.

In order to evaluate the effectiveness of SPINNAKER predictions, we exploited the comparison study conducted in a recent review article [11], where the authors compared the methodology implemented by SPINNAKER [13] with respect to other computational methods, when applied to breast cancer dataset. In particular, to conduct the comparison, they chose to focus on statistics-based methods, since approaches based on mathematical modelling only quantitatively describe a minimum number of ceRNA interaction network, and hence they can be only used to investigate a small number of ceRNA interactions. Among the statistics-based methods, they selected as representative those which are based on Conditional Mutual Information (CMI), Positive Correlation (PC), and Partial Pearson Correlation (PPC) [11]. These methods, as SPINNAKER, encompass two principal parts: computing the candidate ceRNA interactions; assessing the ceRNA interactions. The effectiveness of these methods was evaluated in terms of their ability to recover breast cancer related and/or confirmed sponge interactions by exploiting publicly available databases of computationally predicted and/or experimentally validated ceRNA interactions (Additional file 1: Table S3).

Specifically, the authors considered breast cancer related ceRNA interactions, the ones where the two ceRNAs are genes associated to breast cancer, according to the broad range of experimentally validated databases. CMI predicted a total of 509,504 sponge interactions, among which 20,082 associated to breast cancer and 5 of them experimentally validated. PC method predicted a total of 1,274,708 sponge interactions, among which 46,716 associated to breast cancer and 7 of them experimentally validated. PPC method predicted a total of 702,029 sponge interactions, with 26,803 interactions associated to breast cancer, but no one was experimentally confirmed. The model implemented by SPINNAKER predicted a total of 99,662 sponge interactions, with 4223 associated to breast cancer and 2 of them experimentally confirmed in breast cancer (CNOT6L-PTEN and ZEB2-PTEN). Thus, from this comparative analysis [11], SPINNAKER revealed the highest percentage of identified ceRNA interactions related to breast invasive carcinoma, then resulting as the best method (Fig. 1). It is worth noting that the total number of interactions predicted by SPINNAKER is much lower than other methods shown in the table of Fig. 1. This due to the fact that the rationale behind SPINNAKER methodology is to decrease the number of false positive values as much as possible, by using very high thresholds both in the data collection and processing and ceRNA network building module. This strategy resulted in the higher percentage of disease-specific interactions, as witnessed by the comparison between SPINNAKER and other statistical methods when applied to breast carcinoma, and by the comparison with other more recently developed methods for studying various diseases [21–27]. In Table 1, the comparison between SPINNAKER and two of these methods [21, 22] is shown for thyroid carcinoma (thca). Once again, SPINNAKER resulted the best one in predicting the higher percentage of thyroid carcinoma related genes acting as ceRNAs (Additional file 3).

**Table 1:** Results comparison among SPINNAKER and other two statistical-based methods when applied on thyroid carcinoma (thca) dataset from TCGA

method	player	# total	# thca associated	% thca associated
SPINNAKER	lncRNA	22	6	27
	mRNA	271	39	14
	miRNA	57	29	51
	**Total**	350	74	**21**
Zhao et al., Oncology Reports 2018 [21]	lncRNA	45	3	7
	mRNA	86	17	20
	miRNA	13	8	61
	**Total**	144	28	**19**
Jiang et al., Medicine 2020 [22]	lncRNA	30	2	7
	mRNA	126	15	12
	miRNA	11	6	54
	**Total**	167	23	**14**

### Experimentally validated interactions

The most significant prediction of the computational model implemented by SPINNAKER was the discovery of lncRNA PVT1 acting as ceRNA in breast invasive carcinoma (brca) dataset, where it antagonized the miR-200 family to regulate the expression of several messenger RNAs [13]. This finding was confirmed by applying SPINNAKER on the up-to-date TCGA brca dataset with an increased number of patients as in the original paper (Additional file 4) [13]. As further proof of the reliability of our methodology, this result was experimentally validated in a recent research study conducted in non-small cell lung cancer cells, where the authors showed that PVT1 facilitates the tumor invasion functioning as ceRNA to regulate the MMP9 expression via the competitively binding of miR-200 family [7].

### Performance evaluation

In order to quantify the speed-up reached by SPINNAKER implementation, we compared the elapsed times of running it on MALTAB and R platforms, when applied to brca dataset [13]. We observed that SPINNAKER is from 5x (Module 1) up to 100x (Module 2) faster than its ancestor running on MATLAB (Table 2). Furthermore, we checked that all the results obtained by running the original code (MATLAB-based) were confirmed by SPINNAKER (R-based), when applied to the original brca dataset (Additional file 5). We can conclude that the R-based solution can lead to gain a significant amount of time, thus greatly affecting the whole analysis process.

**Table 2:** SPINNAKER elapsed time (in seconds) when running on R (version 4.0.4) and MATLAB (R2014a) in Windows 10 Pro 20H2 19042.928, CPU 11th Gen Intel(R) Core(TM) i7-1185G7 @ 3.00 GHz, RAM:16.0 GB

SPINNAKER	R (s)	MATLAB (s)	Ratio
Module 1	25	120	1/5
Module 2	1184 (~ 20 min)	86,400 (~ 24 h)	1/100

## Conclusions

In this study, we presented SPINNAKER (SPongeINteractionNetworkmAKER), the R implementation and open-source version of a widely established mathematical model that we published for identifying putative competing endogenous RNA (ceRNA) interactions. According to a recent review [11], the methodology implemented by SPINNAKER resulted as the best one in terms of the percentage of discovered ceRNA interactions associated with breast invasive carcinoma. However, the main limitation towards a broader usability of this methodology is that it was developed in MATLAB®, a proprietary programming environment requiring a paid license for installing, operating, and running the software. To solve this issue, SPINNAKER came as an R-based, open-source, simplified, and improved version with respect to the original MATLAB-based implementation, ensuring a greater efficiency by speeding up the whole process of several orders of magnitude. By comparing SPINNAKER with other statistical-based methods, once again it resulted as the best one in terms of higher percentage of disease-associated genes acting as ceRNAs when applied to thyroid carcinoma dataset.

## Availability and requirements

**Project name:** SPINNAKER

**Operating system(s):** Windows 10 Pro, Ubuntu 20.04.3 LTS, macOS High Sierra 10.13.6

**Programming language**: R

**Project page:** https://github.com/sportingCode/SPINNAKER.git

**Other requirements:** R version 3.5.1, R 4.1.2 or higher

**License:** GNU AFFERO GENERAL PUBLIC LICENSE

**Any restrictions to use by non-academics:** license needed

## Supplementary Information

**Additional file 1:** It includes the Figure S1 and Tables S1–S3.

**Additional file 2:** Performance comparison between the original code (MATLAB) versus SPINNAKER (R).

**Additional file 3:** ceRNA interaction network obtained by running SPINNAKER on thyroid carcinoma dataset.

**Additional file 4:** ceRNA interaction network obtained by running SPINNAKER on breast cancer dataset.

**Additional file 5:** Results comparison between the original code (MATLAB) versus SPINNAKER (R).

## Data Availability

SPINNAKER source code is freely available at https://github.com/sportingCode/SPINNAKER.git along with a comprehensive PPT-based guideline. In order to help users get the key points more conveniently, also a practical R-styled plain-text guideline is provided. Finally, a short movie is available to help the user to set the own directory, properly.

## Abbreviations

ceRNA: Competing endogenous RNA
SPINNAKER: SPongeINteractionNetworkmAKER
lncRNAs: Long non-coding RNAs
brca: Breast invasive carcinoma
thca: Thyroid carcinoma
TCGA: The Cancer Genome Atlas
miRNAs: MicroRNAs
