# Supplementary material for: SPINNAKER: an R-based tool to highlight key RNA interactions in complex biological networks
Source: BMC Bioinformatics. 2022 May 6;23:166. doi: 10.1186/s12859-022-04695-x (PMC9073480; doi:10.1186/s12859-022-04695-x)
Supplement: Supplementary file 1 — Additional file 1: It includes the Figure S1 and Tables S1–S3. [file 12859_2022_4695_MOESM1_ESM.pdf]

Supplementary Figure

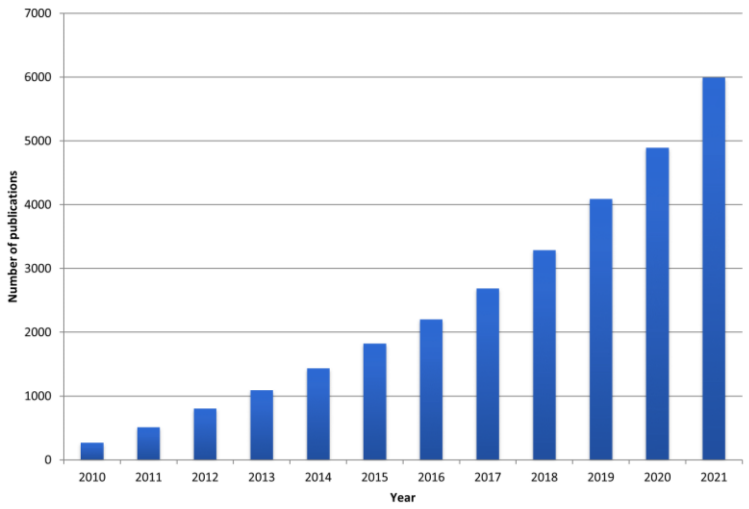

Supplementary Fig. 1. Number of ceRNA-related publications across 2010-2021. The figure shows the number of articles published by year obtained by querying the NCBI PubMed database with the specific keywords: "competing endogenous RNA", "ceRNA", "sponge", “competing triplets” or “RNA-RNA crosstalk”.

Supplementary Tables

Supplementary Table 1. Summary of statistics-based methods.

Name, Year	Method	Data type	Database	Cases of study
Hermes, 2011 [1]	Mutual information and conditional mutual information	Expression data, miRNA-target interaction data	TCGA	Glioblastoma multiforme
Zhou et al., 2014 [2]	Pair-wise correlation	Expression data, miRNA-target interaction data	GEO, TCGA, miRanda	Breast invasive carcinoma
Paci et al., 2014 [3]	Sensitivity correlation	Expression data, miRNA-target interaction data	TargetScan, miRTarBase, TCGA	Breast invasive carcinoma
Xu et al., 2015 [4]	Pair-wise correlation	Expression data, miRNA-target interaction data, cancer-associated miRNAs	TCGA, TargetScan, miRanda, PITA, starBase V2.0, MsigDB	Multi-cancer analysis
Zhang et al., 2016 [5]	Establish significance to sensitivity correlation measure [3]	Expression-data, miRNA-target interaction data	TargetScan, miRanda, PITA, RNAhybrid, DIANA-TarBase, mirTarBase, TCGA	Multi-cancer analysis
Cernia, 2017 [7]	Scoring function for ceRNA prediction, SVM for ceRNA classification	Expression-data, miRNA-target interaction data	miRecords, starBase v2.0, miRTarBase, CLASH, TCGA	Multi-cancer analysis
Cancerin, 2018 [8]	LASSO-based method [9], sensitivity correlation	Expression-data, miRNA-target interaction data, Copy number alteration, DNA methylation	starBase v2.0, TargetScan, miRTarBase, lnCeDB, DIANA-LncBase v2, TCGA	Multi-cancer analysis
SPONGE, 2019 [10]	multiple miRNA sensitivity correlation	Expression-data, miRNA-target interaction data	TargetScan, miRcode, miRTarBase, DIANA-LncBase v2, TCGA	Multi-cancer analysis

Supplementary Table 2. Summary of mathematical modelling methods.

Name, Year	Method	Data type	Database	Case of study
Figliuzzi et al. 2013 [11]	deterministic model	literature model parameters, miRNA-target interaction data	---	Simulated data
Ala et al., 2013 [12]	deterministic model	literature model parameters, miRNA-target interaction data	TargetScan	Simulated data
Chiu et al. 2018 [13].	deterministic model	Expression data, literature model parameters, miRNA-target interaction data	TCGA	Multi-cancer analysis
Miotto et al., 2019 [14]	deterministic model	literature model parameters, miRNA-target interaction data	CLASH interactome	Simulated data
Bosia et al., 2013 [15]	stochastic model	literature model parameters, miRNA-target interaction data	---	Simulated data

Supplementary Table 3. Summary of the main databases of ceRNA interactions.

Database	Type	Link	Last Release
ceRDB [16]	Computational prediction	https://www.oncomir.umn.edu/cefinder/	2011
lnCeDB [17]	Computational prediction/ Experimental validation	http://gyanxet-beta.com/lncedb/index.php	2014
spongeScan [18]	Computational prediction	http://spongescan.rc.ufl.edu	2016
miRSponge [19]	Experimental validation	http://bio-bigdata.hrbmu.edu.cn/miRSponge/	2015
lncACTdb 2.0 [20]	Computational prediction/ Experimental validation	http://www.bio-bigdata.net/LncACTdb/	2018
ENCORI (previously starBase 2.0 [21])	Computational prediction/ Experimental validation	http://starbase.sysu.edu.cn/	2019

References

[1] Sumazin P, Yang X, Chiu H-S, et al. An extensive microRNA-mediated network of RNA-RNA interactions regulates established oncogenic pathways in glioblastoma. *Cell* 2011; 147: 370–381.

[2] Zhou X, Liu J, Wang W. Construction and investigation of breast-cancer-specific ceRNA network based on the mRNA and miRNA expression data. *IET Syst Biol* 2014; 8: 96–103.

[3] Paci P, Colombo T, Farina L. Computational analysis identifies a sponge interaction network between long non-coding RNAs and messenger RNAs in human breast cancer. *BMC Syst Biol* 2014; 8: 83.

[4] Xu J, Li Y, Lu J, et al. The mRNA related ceRNA-ceRNA landscape and significance across 20 major cancer types. *Nucleic Acids Res* 2015; 43: 8169–8182.

[5] Zhang Y, Xu Y, Feng L, et al. Comprehensive characterization of lncRNA-mRNA related ceRNA network across 12 major cancers. *Oncotarget* 2016; 7: 64148–64167.

[6] Wang J-B, Liu F-H, Chen J-H, et al. Identifying survival-associated modules from the dysregulated triplet network in glioblastoma multiforme. *J Cancer Res Clin Oncol* 2017; 143: 661–671.

[7] Sardina DS, Alaimo S, Ferro A, et al. A novel computational method for inferring competing endogenous interactions. *Brief Bioinform* 2017; 18: 1071–1081.

[8] Do D, Bozdag S. Cancerin: A computational pipeline to infer cancer-associated ceRNA interaction networks. *PLoS Comput Biol* 2018; 14: e1006318.

[9] Tibshirani : The lasso problem and uniqueness, https://projecteuclid.org/euclid.ejs/1369148600 (accessed 22 January 2020).

[10] List M, Dehghani Amirabad A, Kostka D, et al. Large-scale inference of competing endogenous RNA networks with sparse partial correlation. *Bioinforma Oxf Engl* 2019; 35: i596–i604.

[11] Figliuzzi M, Marinari E, De Martino A. MicroRNAs as a selective channel of communication between competing RNAs: a steady-state theory. *Biophys J* 2013; 104: 1203–1213.

[12] Ala U, Karreth FA, Bosia C, et al. Integrated transcriptional and competitive endogenous RNA networks are cross-regulated in permissive molecular environments. *Proc Natl Acad Sci* 2013; 110: 7154–7159.

[13] Chiu H-S, Martínez MR, Komissarova EV, et al. The number of titrated microRNA species dictates ceRNA regulation. *Nucleic Acids Res* 2018; 46: 4354–4369.

[14] Miotto M, Marinari E, De Martino A. Competing endogenous RNA crosstalk at system level. *PLoS Comput Biol* 2019; 15: e1007474.

[15] Bosia C, Pagnani A, Zecchina R. Modelling competing endogenous RNA networks. *PLoS One* 2013; 8: e66609.

[16] Sarver AL, Subramanian S. Competing endogenous RNA database. *Bioinformation* 2012; 8: 731–733.

[17] Das S, Ghosal S, Sen R, et al. lnCeDB: Database of Human Long Noncoding RNA Acting as Competing Endogenous RNA. *PLoS ONE*; 9. Epub ahead of print 13 June 2014. DOI: 10.1371/journal.pone.0098965.

[18] Furió-Tarí P, Tarazona S, Gabaldón T, et al. spongeScan: A web for detecting microRNA binding elements in lncRNA sequences. *Nucleic Acids Res* 2016; 44: W176-180.

[19] Wang P, Zhi H, Zhang Y, et al. MiRSponge: A manually curated database for experimentally supported miRNA sponges and ceRNAs. *Database*; 2015. Epub ahead of print 2015. DOI: 10.1093/database/bav098.

[20] Wang P, Li X, Gao Y, et al. LncACTdb 2.0: an updated database of experimentally supported ceRNA interactions curated from low- and high-throughput experiments. *Nucleic Acids Res* 2019; 47: D121–D127.

[21] Li J-H, Liu S, Zhou H, et al. starBase v2. 0: decoding miRNA-ceRNA, miRNA-ncRNA and protein–RNA interaction networks from large-scale CLIP-Seq data. *Nucleic Acids Res* 2013; gkt1248.
