# Supplementary material for: SPINNAKER: an R-based tool to highlight key RNA interactions in complex biological networks
Source: BMC Bioinformatics. 2022 May 6;23:166. doi: 10.1186/s12859-022-04695-x (PMC9073480; doi:10.1186/s12859-022-04695-x)
Supplement: Supplementary file 2 — Additional file 2: Performance comparison between the original code (MATLAB) versus SPINNAKER (R). [file 12859_2022_4695_MOESM2_ESM.pdf]

Original code (MATLAB) *versus* SPINNAKER (R)

Performance comparison

In the following, we detailed the additional features of SPINNAKER running on R with respect to the original version running on MATLAB, together with the implementation differences determining the speed-up of the R solution with respect to the MATLAB one.

Additional features

- Unlike MATLAB version, SPINNAKER drastically simplifies the *data collection step* by:

    a. <u>drastically reducing the number of input files:</u>
    - In the original code (MATLAB), the user has to provide <u>5 input data matrices</u>: (i) protein-coding RNAs (mRNAs) expression levels (candidate ceRNA 1), (ii) long non-coding RNAs (lncRNAs) expression levels (candidate ceRNA 2), (iii) miRNAs expression levels, (iv) miRNA-mRNA target interactions, (v) miRNA-lncRNA target interactions.
    - In SPINNAKER, the user has to provide <u>2 input data matrices</u>: (i) RNAs expression levels, including both coding and non-coding RNAs, (ii) miRNAs expression levels.

    b. <u>automatically performing the input data matrices creation:</u>
    - SPINNAKER automatically queries NCBI's Gene database, separates the candidate ceRNA in the two classes of ceRNAs selected by the user (e.g., mRNAs versus lncRNAs), and creates the expression data matrix for each ceRNA type.
    - SPINNAKER automatically queries TargetScan and miRWalk databases to retrieve the miRNA-target interactions for mRNAs and for lncRNAs, respectively, and creates the two miRNA-target interaction matrices.

    These improvements operated by SPINNAKER have the advantage of lightening the unexpert user's task by saving him a lot of hard scripting and at the same time guaranteeing to have always up-to-date information.

- Unlike MATLAB version that only searched for lncRNAs acting as ceRNAs, SPINNAKER offers the possibility of considering different RNA types acting as ceRNAs.
- Unlike MATLAB version, SPINNAKER presents a *modular structure* that allowed to:

    a. <u>Save the results after the execution of each module is completed.</u>

    In the MATLAB version, all the files and plots are saved at the end of the run and thus if the user want to change some parameters, as well as encounter an error, he/she has to restart the analysis from the beginning losing all the results and the plots.

b. Start the analysis from any module without necessarily restarting from the beginning every time.

If the user wants to change the Pearson correlation or sensitivity threshold, he/she can load the files saved after the execution of Module 1 and thus restart directly from Module 2.

c. Save disk space.

In the MATLAB version, together with the .png files also the .fig files are saved to allow the user to change the plot features when required without running again the code. In the R version, only the .pdf files of each figure are saved. In fact, since the output files of each module are saved at each step, if the user wants to change some figure settings during the code execution, he/she can just upload the saved files and run Module 2 that creates the plot of interest and change whatever he/she wants.

- Unlike MATLAB version, SPINNAKER allows the user to choose if execute or not some parts of the code that are computationally onerous, like the seed-match and statistical analysis. This analysis allows to restrict the number of miRNAs to those ones that have the list of the corresponding ceRNAs enriched in their own targets, and then selects only those triplets with a very high sensitivity correlation.

Speed-up

- The main difference between the original code (MATLAB) and SPINNAKER (R) version, which drastically changes the efficiency of the two solutions, relies on the fact that the MATLAB implementation exploits traditional looping constructs such as the for and while loop, while the R implementation exploits also *apply() functions. In a high-level programming language such as R, it is typically a good idea to avoid loops whenever possible, since looping in high-level programming languages can be very time-consuming. On the contrary, the *apply() functions provide looping functionality using a single function call that would be faster than running through a for loop. This allows to drastically speed-up the entire analysis, with a speed-up that increases with the number of performed loops as shown in Figure S1.

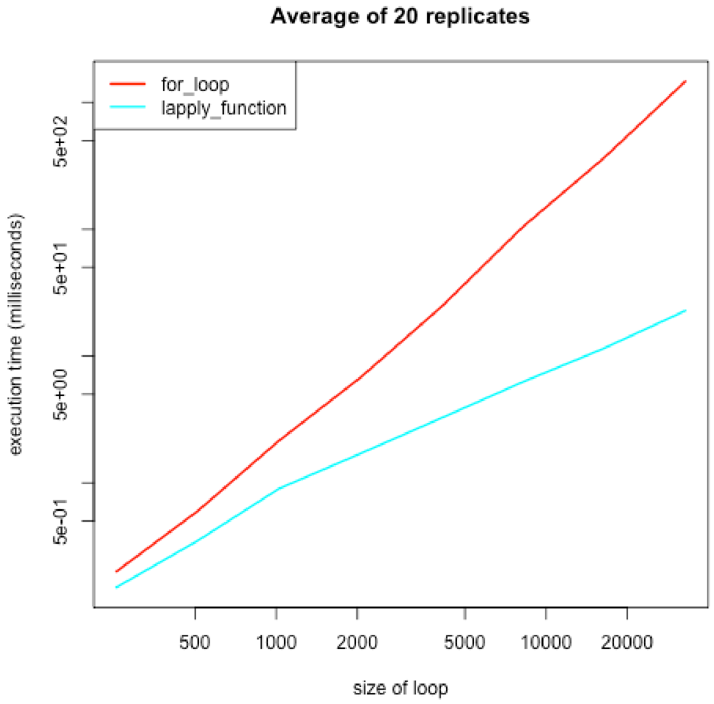

Figure S1. Estimated time of execution of different looping functions. Plot of the execution time of a (trivial) loop that returns a vector of integers 1 to n, for different values of n, using two different variants of running a loop in R: for_loop (red line) and lapply_function (cyan line).
