# Supplementary material for: SPINNAKER: an R-based tool to highlight key RNA interactions in complex biological networks
Source: BMC Bioinformatics. 2022 May 6;23:166. doi: 10.1186/s12859-022-04695-x (PMC9073480; doi:10.1186/s12859-022-04695-x)
Supplement: Supplementary file 5 — Additional file 5: Results comparison between the original code (MATLAB) versus SPINNAKER (R). [file 12859_2022_4695_MOESM5_ESM.pdf]

Original code (MATLAB) *versus* SPINNAKER (R)

Results comparison

We compared the results obtained by using the original code running on MALTAB and SPINNAKER running on R environment, when analysing the original dataset of breast invasive carcinoma from [1], with 20531 genes and 114 samples (72 tumors and 72-matched normal tissues) retrieved from The Cancer Genome Atlas [2].

We found comparable results between the original code running on MATLAB and SPINNAKER running on R that we detailed in the following.

ceRNA network

By running the original code (on MATLAB) and SPINNAKER (on R), we found 92% of common ceRNA-ceRNA-miRNA triplets in breast cancer tissues (Figure S1 left), and 87 % of common triplets in breast normal tissues (Figure S1 right).

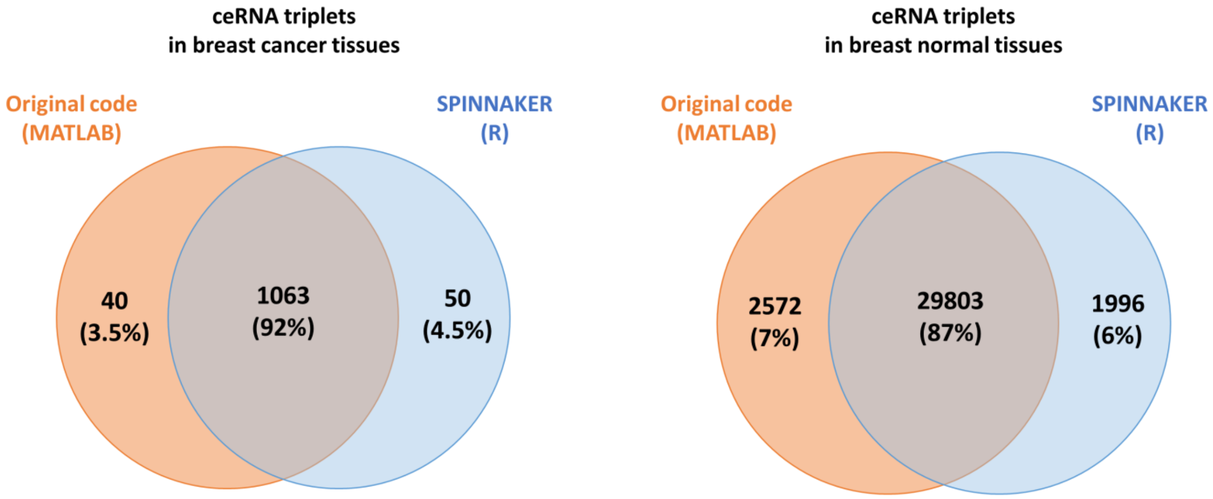

Figure S1. Venn Diagram of triplets (ceRNA-ceRNA-miRNA) obtained from breast cancer (left) and normal (right) tissues by running the original code (on MATLAB) and SPINNAKER (on R).

The corresponding heatmaps of sensitivity correlations are shown in Figure S2-3.

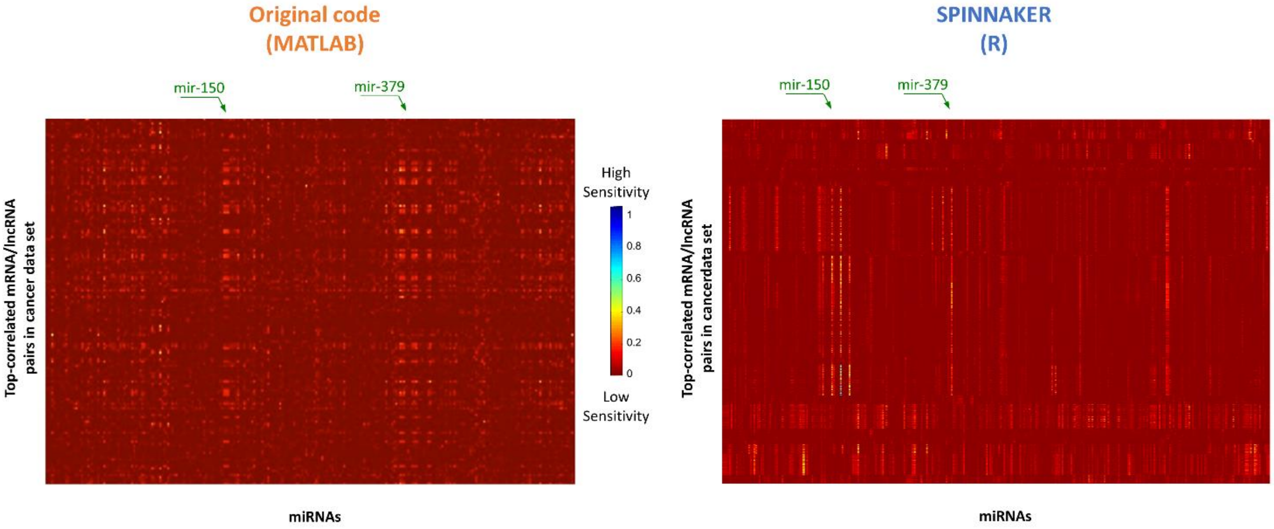

Figure S2. Heatmap of sensitivity correlation, calculated for the top-correlated RNA pairs in the cancer breast dataset by running the original code on MATLAB (left) and SPINNAKER on R (right). Bright vertical stripes refer to a small set of miRNAs mediating the interactions between the top-correlated RNA pairs; sensitivity correlation values (S) increase from red (S=0) to blue (S=1).

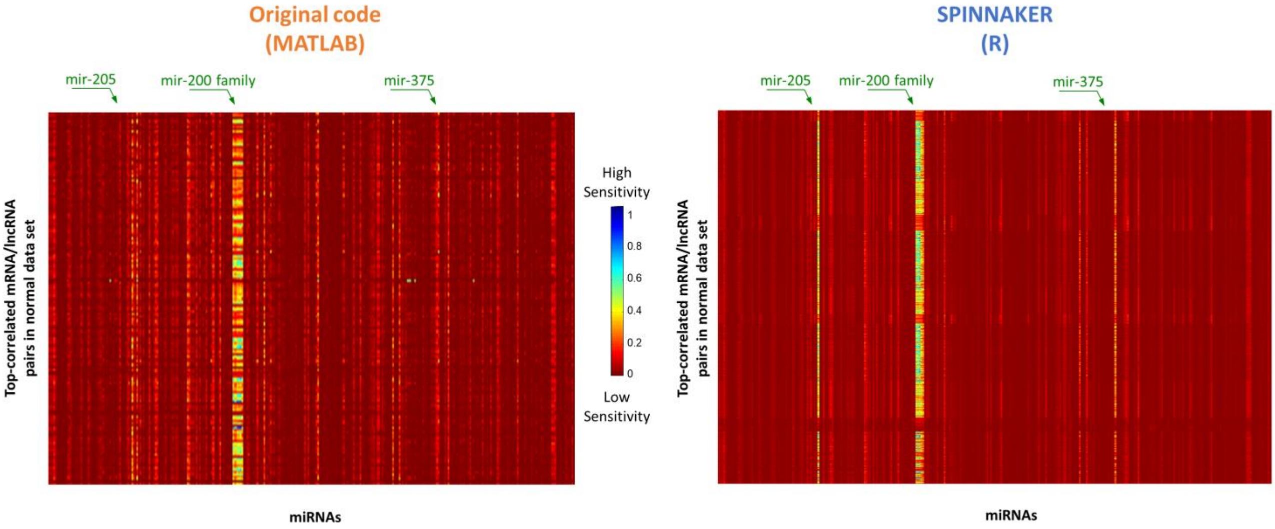

Figure S3. Heatmap of sensitivity correlation, calculated for the top-correlated RNA pairs in the normal breast dataset by running the original code on MATLAB (left) and SPINNAKER on R (right). Bright vertical stripes refer to a small set of miRNAs mediating the interactions between the top-correlated RNA pairs; sensitivity correlation values (S) increase from red (S=0) to blue (S=1).

The detailed list of miRNAs mediating the ceRNA interactions in breast cancer and normal tissues are shown in Table S1 and S2, respectively.

Table S1. miRNAs names along with the number of ceRNA interactions that they are mediating in cancer tissues by running the original code on MATLAB (right) and SPINNAKER on R (left). Common miRNAs are highlighted in red.

Cancer breast tissues

SPINNAKER (R)	
miRNA	# ceRNA mediated interactions
hsa-mir-150	838
hsa-mir-379	116
hsa-mir-370	26
hsa-mir-382	25
hsa-let-7c	22
hsa-mir-93	17
hsa-mir-432	13
hsa-mir-127	12
hsa-mir-106b	8
hsa-mir-199a-1	6
hsa-mir-411	6
hsa-mir-155	5
hsa-mir-146a	4
hsa-mir-223	4
hsa-mir-134	2
hsa-mir-18a	2
hsa-mir-199b	2
hsa-mir-1307	1
hsa-mir-17	1
hsa-mir-22	1
hsa-mir-511-1	1
hsa-mir-889	1
**Total**	**1113**

Original code (MATLAB)	
miRNA	# ceRNA mediated interactions
hsa-mir-150	825
hsa-mir-379	111
hsa-mir-370	26
hsa-mir-382	23
hsa-let-7c	21
hsa-mir-93	17
hsa-mir-411	15
hsa-mir-432	13
hsa-mir-127	12
hsa-mir-106b	8
hsa-mir-155	5
hsa-mir-199a-1	4
hsa-mir-223	4
hsa-mir-134	2
hsa-mir-146a	2
hsa-mir-18a	2
hsa-mir-199b	2
hsa-mir-410	2
hsa-mir-494	2
hsa-mir-1248	1
hsa-mir-1307	1
hsa-mir-17	1
hsa-mir-22	1
hsa-mir-511-1	1
hsa-mir-543	1
hsa-mir-889	1
**Total**	**1103**

Table S2. miRNAs names along with the number of ceRNA interactions that they are mediating in normal tissues by running the original code on MATLAB (right) and SPINNAKER on R (left). Common miRNAs are highlighted in red.

Normal breast tissues

SPINNAKER (R)	
miRNA	# ceRNA mediated interactions
hsa-mir-141	7546
hsa-mir-200a	7430
hsa-mir-200c	4381
hsa-mir-200b	3993
hsa-mir-205	3656
hsa-mir-429	1523
hsa-mir-375	1144
hsa-mir-22	777
hsa-mir-181c	542
hsa-mir-452	315
hsa-mir-378	215
hsa-mir-224	102
hsa-mir-215	86
hsa-mir-652	29
hsa-mir-193a	23
hsa-mir-335	15
hsa-mir-146b	5
hsa-mir-148	5
hsa-mir-181d	5
hsa-mir-196a-1	4
hsa-mir-133a-1	2
hsa-mir-193b	1
**Total**	**31799**

Original code (MATLAB)	
miRNA	# ceRNA mediated interactions
hsa-mir-141	7526
hsa-mir-200a	7410
hsa-mir-200c	4348
hsa-mir-200b	3965
hsa-mir-205	3590
hsa-mir-135b	1504
hsa-mir-375	1076
hsa-mir-31	890
hsa-mir-22	767
hsa-mir-181c	538
hsa-mir-452	315
hsa-mir-378	211
hsa-mir-224	105
hsa-mir-215	86
hsa-mir-652	29
hsa-mir-335	15
**Total**	**32375**

PVT1 sub-network

By extracting the sub-network of ceRNA interactions orchestrated by the long-non coding RNA PVT1 in normal breast tissues, we found that the miR-200 family is the most represented miRNA, mediating 85% of the ceRNA interactions when running the original code (MATLAB) and 89% of the ceRNA interactions when running SPINNAKER (R), followed by the miR-205 in both cases (Figure S4). Thus, the main result of the long non-coding RNA PVT1 functioning as ceRNA for the miR-200 family is confirmed.

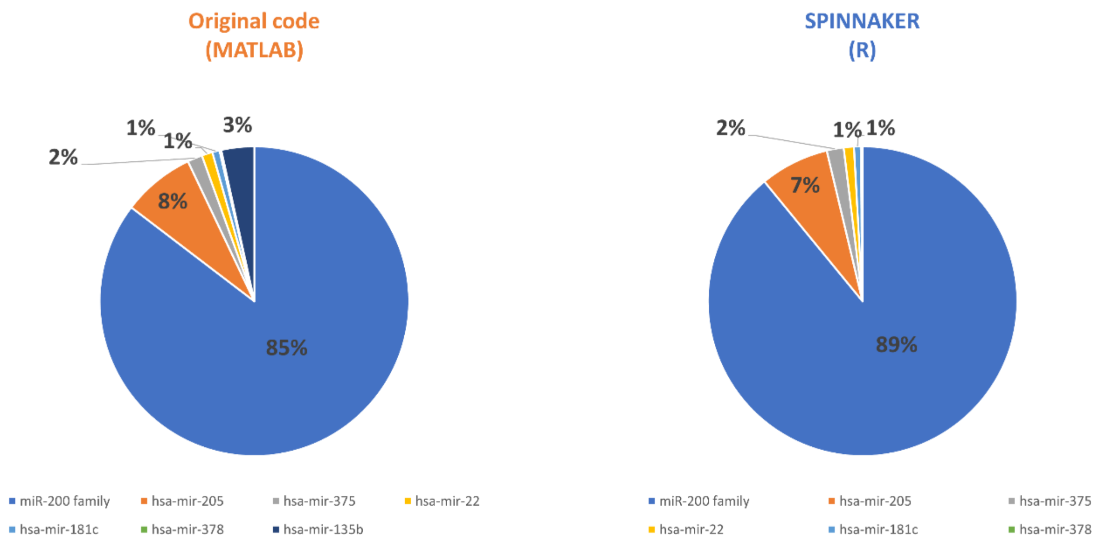

Figure S4. Pie chart of the miRNAs mediating the PVT1-ceRNA interactions in normal breast tissues by running the original code on MATLAB (left) and SPINNAKER on R (right)

Taken together all these findings show that the results obtained by using the original code (MATLAB) are confirmed by SPINNAKER (R).

References

[1] Paci P, Colombo T, Farina L. Computational analysis identifies a sponge interaction network between long non-coding RNAs and messenger RNAs in human breast cancer. *BMC Syst Biol* 2014; 8: 83.

[2] Tomczak K, Czerwinska P, Wiznerowicz M, et al. The Cancer Genome Atlas (TCGA): an immeasurable source of knowledge. *Contemp Oncol Pozn* 2015; 19: A68–A77.
